# Detecting anxiety and depression among infertile patients undergoing IVF-ET using the fertility quality of life and the patient health questionnaire-4 tools

**DOI:** 10.1186/s40359-026-04481-x

**Published:** 2026-04-07

**Authors:** Chih-Wei Lin, Yu-Hsuan Lee, Chen-Yi Yang, Ting-Chien Lin, Yu-Hsien Wu, Huang-Tz Ou, Meng-Hsing Wu, Taiwan HPA FertiQoL Research Task Force, Chen-Lan Kuo, Chia-Ling Wu, Hsin-Yi Yen, Hui-Chen Ko, Kao-Chin Chen, Keng-Shin Lin, Mei-Chou Chen, Meng-Hsing Wu, Yang-Chun Mai, Yu-Yun Hsu

**Affiliations:** 1 Department of Obstetrics and Gynecology, National Cheng Kung University Hospital, College of Medicine, National Cheng Kung University, Tainan, 704 Taiwan; 2 Institute of Clinical Pharmacy and Pharmaceutical Sciences, College of Medicine, National Cheng Kung University, Tainan, 701 Taiwan; 3 Department of Obstetrics and Gynecology, Chi Mei Medical Center, Tainan, 710 Taiwan; 4 School of Pharmacy, College of Medicine, National Cheng Kung University, Tainan, 701 Taiwan; 5 Department of Obstetrics and Gynecology, College of Medicine, National Cheng Kung University, Tainan, 701 Taiwan

**Keywords:** Anxiety Disorders, Depressive Disorder, Infertility, Patient Health Questionnaire, Quality of Life

## Abstract

**Purpose:**

Infertility is associated with considerable psychosocial distress, yet many affected individuals lack formal diagnosis or treatment. The Fertility Quality of Life (FertiQoL) tool is widely used to assess quality of life in infertile populations, while the Patient Health Questionnaire-4 (PHQ-4) is a validated screening tool for anxiety and depression. This study investigated whether FertiQoL could be applied as a screening measure to identify high-risk individuals during in vitro fertilization–embryo transfer (IVF-ET).

**Methods:**

This study enrolled participants (320 patients with 444 valid responses) between February and July 2023 across 11 assisted reproductive technology institutions in Taiwan. FertiQoL was used to evaluate quality of life, and PHQ-4 was used to screen for anxiety and depression. Associations between the two measures were analyzed, and a decision tree model was applied to identify an optimal FertiQoL cutoff score for risk classification.

**Results:**

FertiQoL total scores were significantly and negatively correlated with PHQ-4 outcomes (–0.531 for PHQ-2; − 0.525 for GAD-2). A cutoff FertiQoL score of 63.6 provided satisfactory performance in identifying high-risk individuals, demonstrating sensitivities and specificities of 72% and 76% for anxiety (GAD-2), and 81% and 73% for depression (PHQ-2).

**Conclusions:**

The findings support the clinical utility of FertiQoL as a standardized tool not only for quality of life assessment but also for identifying mental health risks in infertile patients.

**Supplementary Information:**

The online version contains supplementary material available at 10.1186/s40359-026-04481-x.

## Capsule Summary

Fertility Quality of Life (FertiQoL) scores showed a strong correlation with Patient Health Questionnaire-4 (PHQ-4) scores. A total FertiQoL cutoff of 63.6 effectively identified anxiety and depression risks.

## Introduction

There is an increasing number of patients suffering from infertility worldwide [1], a disease that affects the couple’s inability to achieve conception despite regular and unprotected intercourse over 12 months [2]. Infertility-related psychosocial distress often arises during the effort to conceive and continues throughout the stressful journey of fertility treatment [3]. These infertility-related stresses not only bring short-term negative impacts on the trajectory of infertility (e.g., failed treatment, poor quality of life [QoL]) [4] but also affect long-term mental health and well-being of sufferers [5]. The prevalence of psychiatric disorders among patients with infertility ranges from 10.2% to as high as 61.1% [3, 6, 7]. This variability is likely attributable to differences in diagnostic criteria, assessment instruments, cultural contexts, sample selection, and timing of psychiatric evaluation. In Taiwan, the prevalence of psychiatric disorders among women attending an assisted reproduction clinic was reported to be 40.2%, with generalized anxiety disorder (23.2%) and major depressive disorder (17.0%) being the most common [8]. Notably, the majority of those with mental disorders were left undiagnosed and untreated [7]. Hence, the assessment of infertility-related psychosocial distress among infertile patients is imperative to support the provision of timely psychosocial care.

Various international tools for assessing the QoL in infertility treatment are available nowadays. Among them, the Fertility Quality of Life (FertiQoL) has been identified as the most widely used scale [9, 10]. It has shown strong reliability and validity [10] across different contexts/cultures and languages of the versions available. The FertiQoL comprises 6 domains covering emotional, mind-body, relational, and social aspects, as well as treatment aspects consisting of environment and tolerability domains, with a total of 34 items. A previous study of 60 subfertile patients showed that the FertiQoL scores were correlated with anxiety, depression, and helplessness cognitions and that the FertiQoL subdomain scores were lower in the at-risk group identified by the SCREENIVF questionnaire [11], a tool to screen patients undergoing fertility treatments at risk for severe emotional problems. These findings underscore the value of the FertiQoL as a universal tool, not only for assessing QoL but also for identifying potential psychiatric symptoms in infertile patients. A comprehensive assessment of the QoL and psychosocial well-being of infertile patients can aid them in making informed decisions and receiving personalized, holistic support throughout their infertility treatment journey. This evaluation also facilitates timely referrals for psychological interventions when necessary. In addition to traditional assessments, the field is increasingly shifting toward multi-modal artificial intelligence systems that offer personalized, real-time emotional support. This shift highlights a growing trend in reproductive medicine to move beyond static screening toward dynamic digital tools, which show significant potential in bridging the existing gap in psychological care for infertile patients [12, 13].

Given the current lack of a standardized and objective algorithm for triaging infertile patients requiring psychosocial care, this study sought to identify an explicit FertiQoL threshold to detect patients at high risk for anxiety and depression in routine care settings. Furthermore, we propose a clinical algorithm for delivering tailored psychosocial support to infertile patients based on these findings.

## Materials and methods

### Participants

The study was commissioned by the Health Promotion Administration, Ministry of Health and Welfare, Taiwan, and enrolled infertile patients attending 11 assisted reproductive technology institutions in Taiwan from February 1 to July 31, 2023. The recruitment and subsequent analysis were conducted at the individual patient level.

### Ethics approval and consent to participate

Psychosocial assessment was routinely performed before the initiation of IVF treatment, and informed consent to the procedures had been obtained from all participants. This study utilized and analyzed data derived from standard clinical practice, without any additional interventions or collection of new patient information. The study was reviewed and approved with the exemption by the Institutional Review Board of National Cheng Kung University Hospital on May 18, 2023 (IRB Number: A-EX-112-013). The study adhered to the World Medical Association Declaration of Helsinki.

### Quality of life assessment

The FertiQoL questionnaire was administered to participants at various stages of the IVF process, including prior to treatment initiation, oocyte retrieval, embryo transfer (ET), or pregnancy testing. The 34 items across 6 subdomains of the FertiQoL are scored on a 5-point scale. By summing the scores of the items, a total score ranging from 0 to 100 can be calculated, with higher scores indicative of better overall QoL. Individual subscale scores can also be derived for each of the 4 domains (emotional, mind/body, relational, social) in the core module and the 2 domains (tolerability, environment) in the treatment module.

### Assessment of anxiety and depressive disorders

To screen the study participants for anxiety and depressive disorders, the 4-item Patient Health Questionnaire (PHQ-4) was administered [14]. The PHQ-4 combines two well-established screening tools: the 2-item Patient Health Questionnaire (PHQ-2) and the 2-item Generalized Anxiety Disorder Scale (GAD-2). Each item on the PHQ-4 is scored on a 4-point scale, ranging from 0 (not at all) to 3 (nearly every day). A score of 3 or higher on either the PHQ-2 or the GAD-2 subscales has been identified as the appropriate cut-off point for indicating the potential presence of depressive or anxiety disorders, respectively. The PHQ-4 was selected for this study due to its brevity, ease of administration, and established validity as an ultra-brief screening tool for anxiety and depressive disorders in various medical and general populations. Its feasibility and accuracy have also been validated in reproductive medicine settings, supporting its use for the early identification of psychological comorbidities in infertility care [15].

### Statistical analyses

The baseline and clinical characteristics of the participants were tabulated, with continuous values, including FertiQoL scores, presented as means with standard deviations (SDs), and categorical values expressed as frequencies with percentages. To further evaluate the association between FertiQoL scores and depressive or anxiety disorders (i.e., PHQ-2 or GAD-2), we applied linear mixed-effects models to estimate mean differences in FertiQoL between participants with PHQ-2 or GAD-2 scores ≥ 3 and those with scores < 3. In these models, subject ID was specified as a random intercept, while PHQ-2 or GAD-2 category was included as a fixed effect without additional covariate adjustment.

Association between the FertiQoL (i.e., subdomains and total) and PHQ-4 (i.e., PHQ-2 and GAD-2) scores was estimated using Pearson’s correlations. To identify the optimal cut-off value of the FertiQoL questionnaire as a means to pinpoint patients with elevated levels of depression and anxiety presented with high PHQ-4 scores, machine learning models, decision tree model analyses [16], were utilized to assess their predictive capabilities. In brief, the decision tree regression model was developed using the scikit-learn package in Python. To evaluate model robustness and reduce the risk of overfitting, internal validation was conducted using 3-fold cross-validation, with mean squared error used as the performance criterion for model optimization. After model development, the final optimized model was evaluated on an independent test set to assess its generalizability. The decision tree model was selected for its ability to handle complex, multivariate interactions. This is particularly relevant for the FertiQoL tool, as it encompasses multiple distinct subdomains that may interact non-linearly to predict psychological distress. The Youden index and operational ease were employed to evaluate the model performances and determine the optimal clinical solution, in which the Youden index provides a single summary measure that balances sensitivity and specificity [17].

## Results

### Participants’ characteristics

A total of 320 patients with 444 valid questionnaire responses (collected before the commencement of the IVF cycle [56.1%], oocyte retrieval [12.4%], ET [21.8%], and pregnancy testing [9.7%]) were included from 11 assisted reproductive technology institutions in Taiwan during the study period, with the majority as female (63.13%) and having female factors (28.75%), and a mean age of 38.06 (± 5.32) years old and infertility duration of 3.29 (± 2.77) years (Table 1).

**Table 1:** Baseline characteristics of study participants stratified by the PHQ-2 and GAD-2 scores above or below the clinical threshold (i.e., 3)

	Overall patients (*n* = 320)		PHQ-2			GAD-2			
			Score ≥3 (*n* = 18)	Score< 3 (*n* = 302)		Score ≥ 3 (*n* = 39)	Score < 3 (*n* = 281)		
Characteristics	No. patients	Mean (sd) or %	Mean (sd) or %		Mean (sd) or %	Mean (sd) or %		Mean (sd) or %	
Age (years)	278	38.06 (5.32)	117 (5.04)		38.13 (5.34)	37.79 (6.19)		38.10 (5.20)	
Female	202	63.13%	77.78%		62.25%	58.97%		63.70%	
BMI (kg/㎡)	229	23.80 (4.39)	20.99 (2.19)^*^		24.01 (4.45)	22.24 (4.43)^*^		24.01 (4.36)	
AMH (ng/mL)	162	2.82 (2.63)	3.32 (4.18)		2.77 (2.45)	3.25 (2.34)		2.75 (2.68)	
Gravida									
Yes	84	26.25%	33.33%		25.83%	25.64%		26.33%	
No	94	29.38%	44.44%		28.48%	28.21%		29.54%	
Missing	142	44.38%	22.22%		45.70%	46.15%		44.13%	
Parity									
Yes	46	14.38%	11.11%		14.57%	5.13%		15.66%	
No	132	41.25%	66.67%		39.74%	48.72%		40.21%	
Missing	142	44.38%	22.22%		45.70%	46.15%		44.13%	
Infertility duration (years)	178	3.29 (2.77)	3.94 (3.67)		3.24 (2.69)	4.14 (3.23)		3.18 (2.70)	
ET failure history									
None	108	33.75%	55.56%		32.45%	33.33%		33.81%	
At least one	70	21.88%	22.22%		21.85%	20.51%		22.06%	
Missing	142	44.38%	22.22%		45.70%	46.15%		44.13%	
Pregnancy loss history									
None	128	40.00%	50.00%		39.40%	28.21%		41.64%	
At least one	50	15.63%	27.78%		14.90%	25.64%		14.23%	
Missing	142	44.38%	22.22%		45.70%	46.15%		44.13%	
Infertility factor									
Male	31	9.69%	11.11%		9.60%	15.38%		8.90%	
Female	92	28.75%	44.44%		27.81%	25.64%		29.18%	
Both	32	10.00%	11.11%		9.93%	10.26%		9.96%	
Unexplained	26	8.13%	11.11%		7.95%	7.69%		8.19%	
Missing	139	43.44%	22.22%		44.70%	41.03%		43.77%	
Type of embryo transfer^a^									
Fresh	12	11.76%	0.00%		12.50%	0.00%		14.12%	
Frozen-thawed	57	55.88%	100.00%		53.13%	64.71%		54.12%	
Missing	33	32.35%	0.00%		34.38%	35.29%		31.76%	
Day of embryo transferred^a^									
Day 1–3	18	17.65%	0.00%		18.75%	11.76%		18.82%	
Day 4–5	51	50.00%	100.00%		46.88%	52.94%		49.41%	
Missing	33	32.35%	0.00%		34.38%	35.29%		31.76%	
Biochemical pregnancy^a^									
Yes	42	41.18%	50.00%		40.63%	47.06%		40.00%	
No	27	26.47%	50.00%		25.00%	17.65%		28.24%	
Missing	33	32.35%	0.00%		34.38%	35.29%		31.76%	
Clinical pregnancy (≥ 10 weeks) ^a^									
Yes	35	34.31%	50.00%		33.33%	35.29%		34.12%	
No	29	28.43%	50.00%		27.08%	23.53%		29.41%	
Yet to reach at analysis	5	4.90%	0.00%		5.21%	5.88%		4.71%	
Missing	33	32.35%	0.00%		34.38%	35.29%		31.76%	
Ongoing pregnancy (≥ 24 weeks) ^a^									
Yes	26	25.24%	33.33%		25.00%	29.41%		24.71%	
No	31	30.10%	50.00%		29.17%	23.53%		31.76%	
Yet to reach at analysis	11	10.68%	16.67%		10.42%	11.76%		10.59%	
Missing	34	33.00%	0.00%		35.42%	35.29%		32.94%	

*Abbreviations*: *sd* standard deviation, *BMI* Body mass index, *AMH* Anti-Müllerian hormone, *ET* Embryo transfer

^a^ 102 out of 320 participants underwent embryo transfer and had the pregnancy outcomes during the study period

*t-value is significant (*p* < 0.05), calculated using an independent samples t-test

### Correlation between the FertiQoL and PHQ-4 scores

The descriptive results of the mean FertiQoL scores and PHQ-4 scores of the participants are shown in Table 2. Mean FertiQoL scores across all domains were significantly lower in participants with PHQ-2 or GAD-2 scores ≥ 3 compared to those with scores < 3. Linear mixed-effects model analyses yielded similar findings. (Supplementary Table 1).

**Table 2:** Descriptive results of mean FertiQoL and PHQ-4 questionnaire scores

	Overall patients	Subsets of patients with PHQ-2		Subsets of patients with GAD-2	
		Score ≥ 3	Score < 3	Score ≥ 3	Score < 3
**FertiQoL domains/subdomains**	Mean (sd)	Mean (sd)	Mean (sd)	Mean (sd)	Mean (sd)
Emotional	66.9 (18.9)	46.6 (19.6)^*^	68.2 (18.2)	51.1 (20.3)^*^	69.3 (17.6)
Mind/body	72.7 (21.7)	42.6 (27.9)^*^	74.6 (19.8)	50.7 (26.3)^*^	75.9 (18.9)
Relationship	71.0 (13.9)	60.4 (9.3)^*^	71.7 (13.8)	64.3 (11.5)^*^	72.0 (13.9)
Social	71.7 (16.1)	50.8 (15.0)^*^	73.0 (15.3)	58.9 (17.2)^*^	73.6 (15.1)
**Core**	70.6 (15.3)	50.1 (15.3)^*^	71.9 (14.4)	56.2 (16.4)^*^	72.7 (14.0)
Tolerability	68.4 (20.2)	48.8 (24.1)^*^	69.6 (19.3)	54.9 (23.9)^*^	70.4 (18.8)
Environment	71.9 (16.4)	68.3 (15.6)^*^	72.1 (16.4)	66.5 (14.6)^*^	72.6 (16.5)
**Treatment**	70.5 (14.2)	60.5 (11.9)^*^	71.1 (14.1)	61.9 (12.8)^*^	71.7 (13.9)
**Total score**	70.6 (13.9)	53.2 (12.7)^*^	71.6 (13.2)	57.9 (13.7)^*^	72.4 (12.9)

Abbreviations: *sd* standard deviation, *GAD-2* 2-item Generalized Anxiety Disorder, *PHQ-2* 2-item Patient Health Questionnaire

*t-value is significant (*p* < 0.05), calculated using an independent samples t-test

As shown in Table 3, the total FertiQoL scores were highly correlated with the PHQ-2 and GAD-2 scores, with correlation coefficients of -0.531 and − 0.525, respectively (Table 3). Compared to the treatment module, the core FertiQoL showed stronger correlations with the PHQ-4 scores, with correlation coefficients of -0.524 for PHQ-2 and − 0.528 for GAD-2, compared to the treatment FertiQoL, which had correlations of -0.402 for PHQ-2 and − 0.372 for GAD-2 (Table 3).

**Table 3:** Correlation between FertiQoL score, and PHQ-2 and GAD-2 questionnaire scores

Domains/subdomains of FertiQoL	PHQ-2	GAD-2
	Coefficient (95% CIs)^b^	Coefficient (95% CIs)^b^
Core	-0.52 (-0.59, -0.45)^a^	-0.53 (-0.59, -0.46)^a^
Emotional	-0.47 (-0.54, -0.39)^a^	-0.50 (-0.57, -0.43)^a^
Mind/body	-0.49 (-0.57, -0.43)^a^	-0.55 (-0.61, -0.48)^a^
Relationship	-0.35 (-0.43, -0.26)^a^	-0.28 (-0.36, -0.19)^a^
Social	-0.48 (-0.55, -0.40)^a^	-0.45 (-0.52, -0.37)^a^
Treatment	-0.40 (-0.48, -0.32)^a^	-0.37 (-0.45, -0.29)^a^
Tolerability	-0.39 (-0.47, -0.31)^a^	-0.41 (-0.49, -0.33)^a^
Environment	-0.26 (-0.34, -0.17)^a^	-0.19 (-0.29, -0.11)^a^
Total score	-0.53 (-0.59, -0.46)^a^	-0.53 (-0.59, -0.45)^a^

Abbreviations: *PHQ-2* 2-item Patient Health Questionnaire, *GAD-2* 2-item Generalized Anxiety Disorder, *CIs* Confidence intervals

^a^Correlation is significant at the 0.01 level (two-tailed)

^b^The Fisher’s exact test was utilized to construct 95% confidence intervals of the correlation coefficients

### Decision tree model for associating FertiQoL with PHQ-4 scores

**Fig. 1:**
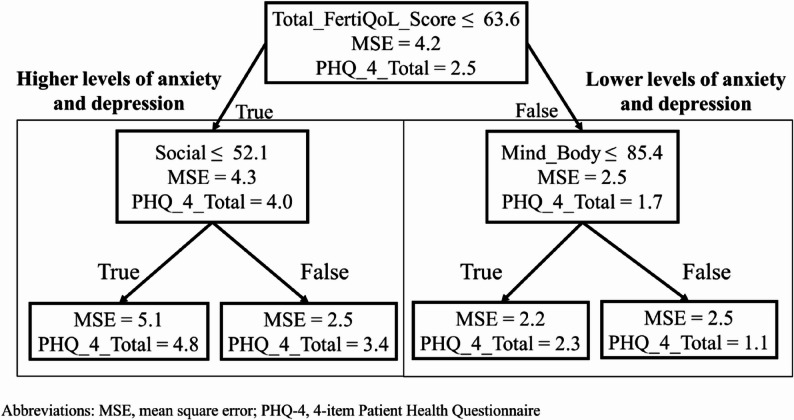
Decision tree for FertiQoL threshold: A decision tree model demonstrating the association between FertiQoL total scores and PHQ-4 outcomes, identifying an optimal threshold score of 63.6 to classify infertile patients at higher risk for anxiety and depression (PHQ-4 **≥** 3). Among these patients (90 participants), those with a social subscale score below 52.1 (40 participants) exhibited even higher PHQ-4 scores compared to those scoring more than 52.1 (50 participants) for social subscale. Abbreviations: MSE, mean square error; PHQ-4, 4-item Patient Health Questionnaire

Figure 1 presents the results of decision tree model analyses that incorporated all FertiQoL sub-domains. An optimal FertiQoL threshold of 63.6 points was identified to classify patients at risk for anxiety or depressive disorders, as determined by the PHQ-4. The model demonstrated sensitivities of 81% and 72% and specificities of 73% and 76% for the PHQ-2 and GAD-2, respectively, with corresponding Youden indices of 0.53 and 0.48 (Table 4). Patients scoring below this threshold had an average PHQ-4 total score of 4.04 (± 2.08), while those above the threshold averaged 1.75 (± 1.59) (Fig. 1).

**Table 4:** Results of Youden index with corresponding sensitivities and specificities for the prediction models with the QoL scores measured by FertiQoL for the risk of anxiety/depressive disorders determined by the PHQ-4

PHQ-4 subscales	Anxiety/depression risk under cut-off of QoL score (by FertiQoL)	Sensitivity with 95% CIs	Specificity 1 with 95% CIs	Youden index	AUC with 95% CIs
GAD-2	50.0	0.35 (0.23, 0.49)	0.95 (0.92, 0.97)	0.30	0.79 (0.65, 0.93)
GAD-2	55.0	0.42 (0.29, 0.56)	0.90 (0.87, 0.93)	0.32	0.77 (0.66, 0.88)
GAD-2	60.0	0.65 (0.51, 0.77)	0.83 (0.79, 0.87)	0.48	0.81 (0.74, 0.88)
**GAD-2**	**63.6**	**0.72 (0.58**,** 0.83)**	**0.76 (0.71**,** 0.80)**	**0.48**	**0.80 (0.74**,** 0.86)**
GAD-2	65.0	0.74 (0.60, 0.84)	0.73 (0.68, 0.77)	0.46	0.79 (0.73, 0.86)
GAD-2	70.0	0.77 (0.64, 0.87)	0.59 (0.54, 0.64)	0.36	0.75 (0.68, 0.81)
GAD-2	75.0	0.84 (0.72, 0.93)	0.44 (0.39, 0.49)	0.28	0.73 (0.64, 0.82)
PHQ-2	50.0	0.46 (0.27, 0.67)	0.94 (0.91, 0.96)	0.40	0.81 (0.70, 0.93)
PHQ-2	55.0	0.54 (0.33, 0.73)	0.89 (0.85, 0.91)	0.42	0.80 (0.71, 0.89)
PHQ-2	60.0	0.73 (0.52, 0.88)	0.80 (0.76, 0.84)	0.53	0.83 (0.77, 0.89)
**PHQ-2**	**63.6**	**0.81 (0.61**,** 0.93)**	**0.73 (0.68**,** 0.77)**	**0.53**	**0.83 (0.77**,** 0.90)**
PHQ-2	65.0	0.81 (0.61, 0.93)	0.70 (0.65, 0.74)	0.50	0.82 (0.75, 0.88)
PHQ-2	70.0	0.88 (0.70, 0.98)	0.57 (0.52, 0.62)	0.46	0.81 (0.73, 0.90)
PHQ-2	75.0	0.96 (0.80, 1.00)	0.42 (0.38, 0.47)	0.38	0.82 (0.69, 0.95)

*Abbreviations*: *GAD-2 *2-item Generalized Anxiety Disorder, *PHQ-2 *2-item Patient Health Questionnaire, *CIs *Confidence intervals.

1. The prediction model adjusted for individual medical institutions, sex, and stages of infertility treatment.

2. The AUC was estimated using the single–time point method proposed by Zhang and Mueller (2005) [18]

## Discussion

To the best of our knowledge, this study is the first to examine the association between the FertiQoL tool—a well-established universal instrument for assessing the QoL among infertile patients—and the PHQ-4 questionnaire, used as a screening tool for high risks of anxiety and depression disorders. We found that lower FertiQoL core domain scores, particularly in the emotional and mind/body subdomains, were correlated with higher PHQ-4 scores. An optimal total FertiQoL cutoff score of 63.6 points was identified to classify individuals at risk for psychological distress. Hence, given its popularity as the QoL measure for infertile populations, the FertiQoL tool also holds the potential as a routine psychosocial assessment for infertile patients to screen individuals for anxiety and depression, thereby enhancing psychosocial care and facilitating appropriate referrals for patients in need of support and intervention for the sufferers.

### Considerable psychological burden to infertile individuals: call for timely assessment of high-risk patients for interventions

Extensive research has established that the prevalence of anxiety and depression is high within the infertile population, with up to 60% of individuals experiencing clinically significant psychiatric symptoms [19–21]. Additional risk factors may include somatic symptoms, poor sleep quality, and higher age for these conditions [22]. Despite the high prevalence of psychological distress during infertility treatment, the majority of patients remain without adequate mental health referrals or sufficient psychosocial support [23]. These findings underscore the urgent need for effective screening and diagnostic protocols to appropriately identify high-risk populations and facilitate the timely referral of patients requiring professional intervention.

A comprehensive review of the FertiQoL tool, encompassing 53 published works, reaffirmed the reliability and validity of this instrument for evaluating QoL among infertile patients. Additionally, the instrument has proven to be a robust measure across various global regions and cultural contexts [24]. A validation study of the FertiQoL tool examined its correlation with anxiety and depression, adopting the Hospital Anxiety and Depression Scale (HADS), which encompasses 14 items, and found a significant negative correlation between FertiQoL scores and HADS scores [25]. Furthermore, it showed that infertile women who had reached the threshold for anxiety and depression based on HADS had significantly lower FertiQoL scores, with an average total score of 58.8 and 51.9 for the thresholds of anxiety and depression, respectively.

In contrast, the present study applied machine learning methods (i.e., decision tree model analyses) to identify a statistically significant cut-off point of FertiQoL (i.e., 63.6) for patients at risk of psychological distress, with satisfactory diagnostic values (i.e., the sensitivity/specificity of 0.72/0.81 and 0.76/0.73 for GAD-2 and PHQ-2, respectively). That is, an infertile individual with a total FertiQoL score below 63.6 would be at risk for anxiety and depression disorders, as assessed by the PHQ-4. Our results proposed a higher threshold than the study above that utilized the HADS tool, which may be attributable to differences in the study population/settings (e.g., Dutch versus Taiwanese) and methodology (e.g., measures for anxiety/depression, and statistical approaches for cut-off points). Nevertheless, we adopt the PHQ-4, a tool already validated for use in infertile populations, owing to its brevity and practicality. As a concise four-item questionnaire, we believe that the PHQ-4 would reduce participants’ burden when administered alongside the FertiQoL, which already comprises 34 items. On the other hand, the cultural context of Taiwan deserves consideration, as mental health stigma remains a significant barrier to accurate screening in many Asian populations. Our FertiQoL-based approach frames distress within the context of quality of life rather than psychopathology; consequently, it may bypass the hesitance patients often experience when completing explicit psychiatric assessments.

### Urgency for enhancing the psychological well-being of infertile patients: a proposed algorithm for tailored psychosocial support strategies during infertility treatment

**Fig. 2:**
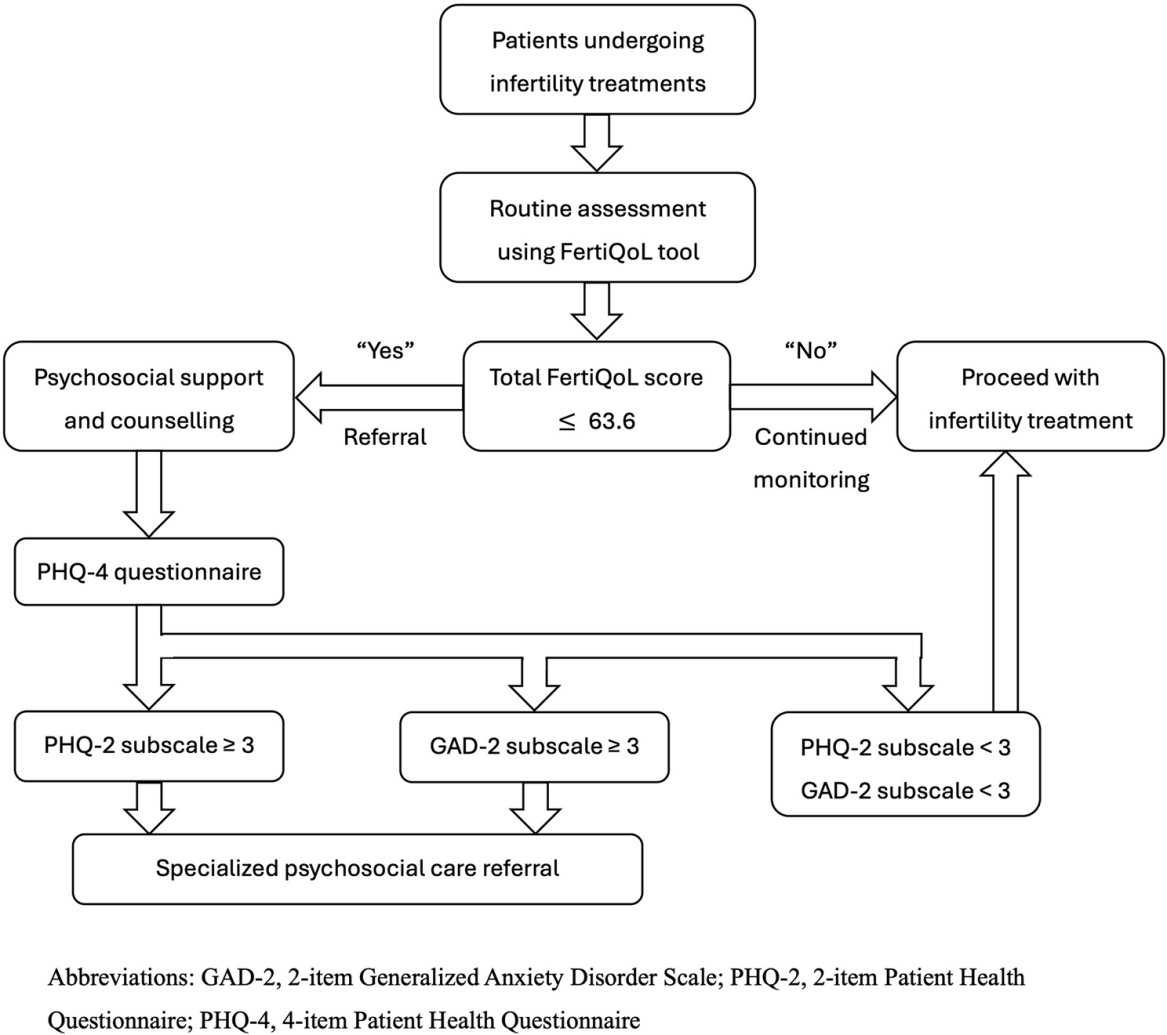
Proposed algorithm of screening depression and anxiety among patients undergoing infertility treatment: A proposed algorithm for delivering tailored psychosocial care to infertile patients undergoing IVF, based on the identified FertiQoL threshold score of 63.6. The algorithm begins with a FertiQoL assessment, followed by a PHQ-4 evaluation for patients scoring below the threshold. Based on PHQ-4 results, patients are triaged into categories requiring either routine monitoring, targeted psychological care, or specialized mental health referrals

According to the guidelines issued by the European Society of Human Reproduction and Embryology (ESHRE) on psychosocial care for infertility patients, fertility staff are recommended to adopt established tools for assessing patients’ needs for psychosocial support. Among these tools, the FertiQoL is a fertility-specific instrument designed to evaluate a wide range of needs and the quality of infertility treatment [26]. On the other hand, some tools, such as the SCREENIVF, can help identify women at risk of emotional maladjustment [27]. While the FertiQoL is not specifically designed to address depression or anxiety disorders, our study underscores its utility in this area by analyzing its association with the PHQ-4 questionnaire with a threshold of the total FertiQoL score that provides diagnostic values for detecting infertile patients for depression/anxiety disorders. Furthermore, as FertiQoL addresses various psychosocial aspects that patients may encounter, we believe that the routine use of FertiQoL can help identify specific psychosocial vulnerabilities tailored by different domains/subdomains of FertiQoL. This will enable fertility staff to focus on these areas and screen for patients who may benefit from professional psychosocial intervention at the same time. Herein, based on our study results, we propose an algorithm for screening depression and anxiety among patients undergoing infertility treatment. The process begins with the universal administration of the FertiQoL questionnaire. For individuals with a total FertiQoL score lower than 63.6, a subsequent screening using the PHQ-4 should be conducted. If the PHQ-4 indicates elevated risk, referral for professional psychosocial care is recommended (Fig. 2). This integrated approach provides a structured pathway for evidence-based care, aligning with international standards to ensure that psychosocial support is systematically delivered and patient-centered.

### Potential limitations

First, the study was conducted exclusively in Taiwan, where universal healthcare coverage is available, which might thus limit the generalizability of the threshold of FertiQoL to other ethnic populations and cultural contexts or healthcare systems. Therefore, although the proposed threshold demonstrated acceptable discriminative performance in this cohort, the FertiQoL cutoff should be regarded as preliminary and exploratory, and external validation in independent populations and diverse clinical settings is required before it can be recommended for routine clinical application. Second, the cross-sectional design of the questionnaire survey may limit the ability to establish causal relationships between impaired QoL and anxiety or depression disorders, and further exploration of the underlying mechanisms driving the correlations between FertiQoL and PHQ-4 scores. Third, although our analyses were conducted at the individual level, we recognize that psychosocial experiences within couples are often interdependent. Nevertheless, because the primary objective of this study was to validate a pragmatic screening threshold for routine clinical practice, the individual level of analysis was considered appropriate for facilitating triage and referral. Future research could employ dyadic analysis to further explore the interplay of QoL between partners. Lastly, due to a lack of comprehensive medical records (e.g., disease diagnoses and prescription drugs) in study data, the mental illnesses a person presents can not be ascertained. However, the score of PHQ-4, a reliable, valid, and cost-effective tool for assessing symptoms related to depression and anxiety in various populations (e.g., pregnancy) [14], might serve as a surrogate indicator. Nevertheless, future longitudinal studies are warranted to corroborate our findings by using clinically confirmed psychiatric diagnoses as the reference standard, thereby enabling an evaluation of the diagnostic accuracy of the FertiQoL cutoff scores.

In conclusion, the FertiQoL, a widely used QoL measure for infertile populations, offers significant clinical utility in identifying high-risk patients with poor psychological well-being. By adopting the proposed threshold and algorithm, clinicians can ensure the delivery of timely and effective mental health support, with interventions specifically tailored to the vulnerable domains identified by the FertiQoL assessment.

## Supplementary Information

Supplementary Material 1: Supplementary Table 1. Estimated mean value of FertiQoL and PHQ-4 questionnaire scores by mixed effect model.

## Data Availability

The data underlying this article cannot be shared publicly for the privacy of individuals that participated in the study. The data will be shared on reasonable request to the corresponding author.
