# Supplementary material for: Detecting anxiety and depression among infertile patients undergoing IVF-ET using the fertility quality of life and the patient health questionnaire-4 tools
Source: BMC Psychol. 2026 Apr 7;14:755. doi: 10.1186/s40359-026-04481-x (PMC13188597; doi:10.1186/s40359-026-04481-x)
Supplement: Supplementary file 1 — Supplementary Material 1: Supplementary Table 1. Estimated mean value of FertiQoL and PHQ-4 questionnaire scores by mixed effect model. [file 40359_2026_4481_MOESM1_ESM.docx]

**Supplementary Table 1: estimated mean value of FertiQoL and PHQ-4 questionnaire scores by mixed effect model**

|  | **Subsets of Patients with PHQ-2** | | | **Subsets of patients with GAD-2** | | |
| --- | --- | --- | --- | --- | --- | --- |
|  | Score≥3 | Score<3 |  | Score≥3 | Score<3 |  |
| **FertiQoL domains/subdomains** | Estimated Mean (SE) | Estimated Mean (SE) | Differences (95% CIs) | Estimated Mean (SE) | Estimated Mean (SE) | Differences (95% CIs) |
| **Core** | 56.68 (2.18) | 71.75 (0.81) | -15.07 (-19.33 ,-10.81)^*^ | 60.95 (1.57) | 72.20 (0.80) | -11.25 (-14.31 ,-8.19)^*^ |
| Emotional | 51.25 (2.96) | 67.74 (1.01) | -16.48 (-22.33 ,-10.64)^*^ | 54.49 (2.09) | 68.44 (1.01) | -13.95 (-18.10 ,-9.79)^*^ |
| Mind/body | 52.92 (3.08) | 74.35 (1.11) | -21.43 (-27.47 ,-15.39)^*^ | 58.09 (2.21) | 75.12 (1.10) | -17.03 (-21.34 ,-12.71)^*^ |
| Relationship | 64.67 (2.30) | 71.89 (0.76) | -7.22 (-11.77 ,-2.66)^*^ | 66.73 (1.63) | 72.11 (0.78) | -5.38 (-8.62 ,-2.14)^*^ |
| Social | 55.34 (2.52) | 73.14 (0.85) | -17.80 (-22.78 ,-12.81)^*^ | 62.17 (1.82) | 73.43 (0.87) | -11.26 (-14.88 ,-7.64)^*^ |
| **Treatment** | 64.21 (2.28) | 71.37 (0.77) | -7.17 (-11.67 ,-2.66)^*^ | 66.00 (1.62) | 71.62 (0.77) | -5.62 (-8.85 ,-2.39)^*^ |
| Tolerability | 58.54 (3.04) | 69.98 (1.07) | -11.43 (-17.41 ,-5.45)^*^ | 63.30 (2.20) | 70.10 (1.08) | -6.80 (-11.12 ,-2.48)^*^ |
| Environment | 68.24 (2.85) | 72.29 (0.90) | -4.06 (-9.72 ,1.61)^*^ | 68.01 (2.00) | 72.61 (0.91) | -4.60 (-8.63 ,-0.56)^*^ |
| **Total score** | 59.15 (1.94) | 71.63 (0.73) | -12.48 (-16.26 ,-8.70)^*^ | 62.75 (1.41) | 71.99 (0.73) | -9.25 (-11.98 ,-6.52)^*^ |

Abbreviations: SE, standard error; GAD-2, 2-item Generalized Anxiety Disorder; PHQ-2, 2-item Patient Health Questionnaire; CIs, confidence intervals.

**p*<0.05, calculated using linear mixed-effects model.
